# ‘People don't get cancer, families do’: Co‐development of a social physical activity intervention for people recently affected by a cancer diagnosis

**DOI:** 10.1111/ecc.13573

**Published:** 2022-03-13

**Authors:** Karen Milton, Karen Poole, Ainslea Cross, Sophie Gasson, Kajal Gokal, Karen Lyons, Richard Pulsford, Andy Jones

**Affiliations:** ^1^ Norwich Medical School University of East Anglia Norwich UK; ^2^ School of Health Sciences University of Surrey Surrey UK; ^3^ School of Psychology University of Derby Derby UK; ^4^ School of Sport, Exercise and Health Sciences Loughborough University Loughborough UK; ^5^ Connell School of Nursing Boston College Newton Massachusetts USA; ^6^ Sport and Health Sciences University of Exeter, St Luke's Campus Exeter UK

**Keywords:** cancer, physical activity, intervention, co‐design, social support

## Abstract

**Objective:**

This research took a co‐design approach to develop a social intervention to support people affected by a cancer diagnosis to be physically active.

**Methods:**

We conducted semi‐structured interviews with five key stakeholder groups: (1) adults with a recent breast or prostate cancer diagnosis; (2) family and friends of cancer patients; (3) healthcare professionals; (4) physical activity providers; and (5) cancer charity representatives. Inductive content analysis was used to identify themes in the data. We then worked with a subset of participants to co‐develop the intervention.

**Results:**

Participants welcomed the idea of a social approach to a physical activity intervention. Input was received on the timing and format of delivery, how to communicate about physical activity to cancer patients and their family and friends and the types of physical activity that would be appropriate. Our findings suggest that interventions need to be flexible in terms of timing and delivery and offer a wide range of physical activity options. These findings directly informed the co‐development of ‘All Together Active’.

**Conclusion:**

All Together Active is designed to support cancer patients and their family and friends to be active throughout treatment and beyond, benefiting their physical and mental health.

## 1 BACKGROUND

In 2020, the World Health Organization (WHO) released the first global physical activity guidelines for people with chronic conditions, including those living with cancer (cancer survivors) (World Health Organization, 2020). The evidence underpinning these guidelines demonstrates that physical activity improves all‐cause mortality, cancer‐specific mortality and risk of cancer recurrence or second primary cancer (Bull et al., 2020). In addition, being active during treatment has been shown to optimise the effects of treatment and reduce side effects, such as fatigue (Kessels et al., 2018). It can also improve mood, concentration and sleep and reduce stress (Campbell et al., 2019). Importantly, the WHO guidelines recognise that even small amounts of activity are beneficial, including light intensity activity, especially if this is in place of sedentary behaviour (Bull et al., 2020).

A key consideration when managing cancer is that the impact is not confined to a diagnosed individual alone but occurs within the context of a social support network of family and friends (Humpel et al., 2007). Some research has investigated the impact of cancer on family caregivers and particularly spouses, but little has examined the effects on the health‐related behaviours, such as smoking, alcohol consumption and physical activity, of others closely associated with the diagnosed patient (Lewis, 2006). Nevertheless, there is evidence that having a strong social network is a significant driver of physical activity in patients with cancer (Barber, 2013; Cummins et al., 2017; Keogh et al., 2014; Macmillan Cancer Support, 2016) and social support involving family and friends is a key component of promoting sustainable, long‐term behaviour change (Carr et al., 2018). Moreover, relational theories including interdependence (Lewis et al., 2006) and communal coping theories (Lyons et al., 1998) purport that the individual with cancer and their care partner are more likely to engage in behaviours (e.g. physical activity) together and experience better physical, mental and relational outcomes when they perceive cancer as a shared experience (Lyons et al., 1998).

Heightened cancer awareness, emotional closeness to another diagnosed with cancer and anxiety about personal vulnerability can create a ‘teachable moment’ (Frazelle & Friend, 2016), which may be a catalyst for concerned family and friends to change their own health‐related behaviours (McBride et al., 2017; Radecki Breitkopf et al., 2014). For example, there is evidence that at least 80% of family members with two or more relatives with colorectal or pancreatic cancer are receptive to taking part in a lifestyle cancer risk reduction programme, even a decade after their relatives have been diagnosed (Howell, Brockman, et al., 2013; Howell, Sinicrope, et al., 2013). Interventions that harness this social cohesion have the potential to promote primary cancer prevention for family and friends (prevention of cancer occurring), in addition to secondary prevention (improved outcomes and reduced risk of recurrence) for those diagnosed with cancer. Yet, there is a dearth of knowledge about the role of the broader social network of family and friends in encouraging and supporting physical activity of the individual with cancer and playing an active role in their own behaviour change. To address this limitation, we utilised a co‐design approach (working collaboratively with end users) to develop a novel social intervention to support people affected by a cancer diagnosis to be physically active. In this paper, we describe this approach and consider the critical role that co‐development plays in the design of public health interventions.

## 2 METHODS

### 2.1 Design

We used a qualitative approach involving interviews with stakeholders and an iterative co‐design process, following the Framework for the Co‐production and Prototyping of Public Health Interventions (Hawkins et al., 2017).

### 2.2 Recruitment

This research involved five key stakeholder groups: (1) adults with a breast or prostate cancer diagnosis within the last 18 months; (2) family and friends of cancer patients; (3) healthcare professionals; (4) physical activity providers; and (5) cancer charity representatives. Healthcare professionals included consultant oncologists, breast cancer nurse practitioners, chemotherapy nurses and a lymphoedema specialist physiotherapist. The physical activity providers had varied qualifications and backgrounds although we did not recruit people based on a minimum level of qualification, rather their experience of working with individuals living with cancer. Qualifications among the group varied and included a degree in sports science, a senior yoga teacher taking referrals from a breast cancer support centre, a Level 4 qualification in cancer exercise and rehabilitation and an associate professor in clinical exercise science. Some providers worked for specific cancer charities, while others worked at community gyms, cancer specific gyms or as freelance exercise instructors. Healthcare professionals, physical activity providers and charity representatives were recruited through the existing networks of the research team and reflected a wide geographical spread across the United Kingdom. Cancer patients and their family and friends were recruited via a local cancer charity (in the region of East Anglia, England).

### 2.3 Procedure

Semi‐structured interviews were conducted by an experienced nurse researcher (SG), either by telephone or face to face, between January and June 2018. The topics covered the facilitators and barriers to physical activity among cancer patients and their family and friends, desirable features of a physical activity intervention for this group and how a social intervention to promote physical activity for both patients and their family and friends could be embedded into cancer care. Interviews were audio‐recorded and lasted for a median of 27 min (range 18–49 min). The recordings were transcribed verbatim and uploaded to the NVivo 12 qualitative software package.

### 2.4 Analysis

Inductive content analysis was used to explore perceptions of the appropriateness of a social intervention to support cancer patients and their family and friends to be physically active, and the key characteristics that such an intervention should include. The interview data were independently coded by three researchers (KP, SG and AC) using thematic analysis (Braun & Clarke, 2006). Coding was undertaken throughout the project, and data saturation was deemed to have been reached when the interviews no longer generated new insights. At that stage, the final codes and themes were agreed between authors.

### 2.5 Intervention development

Participant were invited to join members of the research team to form an intervention development group (IDG), although some felt unable to commit time to this aspect of the research. Through a series of three workshops, and based on the findings from the interviews, the IDG developed an intervention to support people affected by a cancer diagnosis to be physically active. All three workshops were attended by members of the research team, cancer patients and physical activity providers. Some were also attended by healthcare professionals, family and friends of cancer patients and cancer charity representatives.

The workshops were chaired and minuted by research team members, but decision making was shared. Minutes were emailed to those who could not attend to provide an opportunity to contribute to discussions after the event. Workshops began with an overview of the project aims and objectives (i.e. to develop an intervention to promote and support physical activity for people newly diagnosed with cancer and their family and friends). Each workshop was guided by the main themes from our interviews to facilitate and guide a progressive discussion about intervention features. In line with the Framework for the Co‐production and Prototyping of Public Health Interventions, co‐production of the intervention occurred across the series of workshops, where input and refinements were made to the intervention aims, objectives, features and content over time until the final content was agreed by all members of the group. The IDG also designed and worked up prototypes for the intervention materials.

## 3 RESULTS

In total 37 stakeholders were interviewed including patients (*n* = 11, including seven with breast cancer and four with prostate cancer), family and friends (*n* = 7), healthcare professionals (*n* = 6), physical activity providers (*n* = 8) and cancer charity representatives (*n* = 5). It was universally agreed by participants that the impact of a cancer diagnosis is not confined to a diagnosed individual, but is a shared experience as exemplified by the statement ‘People don't get cancer, families do’ (prostate cancer patient). Participants welcomed the idea of a social approach to a physical activity intervention for those recently affected by a cancer diagnosis. It was felt that a social intervention would be more likely to result in successful behaviour change by increasing motivation among diagnosed patients:

If you get somebody who's, you know, in shock, just been diagnosed, can't be bothered to do it and they see their family doing something, it may inspire them to start. (Prostate cancer patient)

I swim with friends, and I'm much more inclined to go swimming than if I just do it by myself. If it's just me it's like oh well, I'll just skip that, but when you've got somebody else there, you're thinking … ‘ooh, I can't let them down’ and then they feel they can't let me down by not going, so you go along anyway. (Charity representative)

(there are) definite links to whether or not that person has a strong support network around them. If they're engaged in a support group … I've found that they're far easier to work with and express that readiness to change a lot easier. If they have a family member local or a friend or are part of a kind of social environment, they're a lot easier to engage with … you tend to expect better results than with individuals that are isolated or perhaps live alone. (Physical activity provider)

It was also felt that undertaking physical activity together would benefit the family and friends. When a family member or friend has been diagnosed with cancer, those around them can feel helpless and unsure how best to show support. A social intervention would benefit family and friends in terms of providing a focussed supportive role in the patient's treatment:

that would then provide family members a very tangible way of helping, wouldn't it, because sometimes you don't really know what to do …. You know, words are very difficult at times, and you can't possibly really know what, you know, what your wife is going through. You think you do, but you can't really know, can you, so anything that you can do to help tangibly I think would be a real positive thing. (Family or friend)

In terms of the delivery of a social intervention to support people affected by a cancer diagnosis to be physically active, five themes were identified from the interview data: (1) timing; (2) format of delivery; (3) framing of communication; (4) types of physical activity; and (5) the importance of progression, enjoyment and flexibility. The findings related to each are summarised below.

### 3.1 Timing

There were mixed views on the appropriate time to introduce the topic of physical activity. Some felt that the point of diagnosis would be appropriate.

Moment of diagnosis … something like that can make you change the way you behave. (Health professional)

I think that it's part of essential information that patients should get at the time of diagnosis, because it's about their physical and mental wellbeing and sometimes about their social interaction. (Charity representative)

However, patients tended to feel that time would be needed to come to terms with the diagnosis before being provided with information on physical activity.

Speaking as a cancer patient, if you'd have said this to me when I'd just been diagnosed with cancer I would have said, ‘Look, I'm too busy to even think about doing that, I'm too busy accepting this into my life’. (Prostate cancer patient)

The importance of timing physical activity promotion around cancer treatment was also emphasised, particularly by the physical activity providers. The importance of being fit, to reduce functional decline throughout treatment, was felt to be a key motivation for patients to get physically active.

… research is showing now that if you can get people to be active … during chemotherapy, or radiotherapy, it does have big benefits of stopping the functional decline, so helps keep the cardiorespiratory fitness up, it helps keep their muscular strength up and it also helps with their confidence, and fatigue as well. (Physical activity provider)

[I have] worked with newly diagnosed patients having chemo. Really enjoyed as beneficial for the patient. Working with the patient early means they don't go through physical deconditioning. (Physical activity provider)

The times immediately following an operation or completion of treatment were also suggested as possible appropriate time points. Overall, we found that there is no single time point that would suit all patients, and the ‘right time’ is likely to be highly variable for different individuals. Therefore, any intervention should be flexible for people to access at different stages along their cancer journey.

### 3.2 Format

The role of the Internet and social media were frequently mentioned, including Apps which recommend different activities in response to how people are feeling and the use of text messages or social media promoting daily challenges.

maybe like a little app or something that goes, ‘How are you feeling today?’, and it's like good, … and then send that, ‘Why don't you, you could try one of these’, and then give them a few options like that … And I guess a text message feels a bit more personal and then they might, prompt them to be like, ‘Actually, that's an idea’. (Family or friend)

Some felt hard copy resources such as leaflets would be particularly appropriate, as they allow the patient to choose when they look at the material, based on when they feel ready. The idea of having an activity diary was mentioned by several participants. Wearable devices and fundraising for cancer charities were also considered to have a role to play in encouraging people to be physically active.

I think coming straight here and talking to people here was quite good and they can give you the information and you can take it home and then once you're in a sort of better place mentally then you can look at it at your own pace. (Breast cancer patient)

So, for some people it's, there's a diary thing, for other people it's about having a challenge, you know, I'm going to do the 5k, you know, for other people it's around raising money for the charity that's helped them. (Charity representative)

It was clear that there is not one format of delivery that will suit everybody and therefore offering a suite of resources in different formats would be needed to maximise the reach of the intervention.

### 3.3 Framing of communication

The importance of providing clear information on physical activity to cancer patients was emphasised, as a diagnosis of cancer can cause uncertainly around what types of physical activities are safe to undertake.

Diagnosis can act as a confidence knock. People are unsure what they can and can't do when it comes to treatment and what sort of exercise they can or cannot do and what sort of side‐effects they will experience. (Physical activity provider)

When communicating the benefits of physical activity, the need to frame messages carefully to avoid instilling a sense of blame was felt to be critical.

‘This can help with recovery, this can help to prevent it’, rather than, … ‘this may have contributed to you getting it’, so telling them in a different way, but you're telling them the same thing, obviously, but just, ‘For the future, this could prevent it’, rather than, ‘It's your fault’. (Family or friend)

We cannot turn around to someone and say ‘actually, totally 100% definitely, you could have prevented this by walking round the block every day’. I mean it's just wrong. (Charity representative)

### 3.4 Types of physical activity

The need for variety was emphasised in relation to the types of physical activity included within the intervention. Providing a range of diverse activities would cater for different interests, as well as ensuring there are activities for all, regardless of the time since diagnosis or their treatment modality and how it is impacting them.

it needs to be a wide range of activities so that everyone's got something. (Breast cancer patient)

Suggestions for activities that can be undertaken at home were considered important as these would be accessible for all and would not require patients to leave the house if they were feeling unwell, unsociable or self‐conscious.

People can do in their own home, because I think the other thing that we get is people, so people who've lost their hair for instance don't want to do exercise with a wig, it's too hot, and you know, it becomes too hot, it becomes impractical throwing your head around. (Charity representative)

The importance of having a broad range of ‘non‐sports’ opportunities was frequently mentioned, and walking was viewed as a particularly appealing and accessible activity. Walking also provides an opportunity to talk, supporting social cohesion, whereas other activities such as fitness classes may be less likely to lend themselves to conversation.

The other great thing about walking with regards these kind of target groups is how social it can be as you're exercising as well. So, there can be a lot of peer support and peer mentoring in these kinds of walks that doesn't come out in a group fitness class. It's a bit more difficult to talk if you're jumping about. (Physical activity provider)

### 3.5 The importance of progression, enjoyment and flexibility

Participants felt that any intervention targeted at cancer patients and their family and friends would need to have three key characteristics: progression, enjoyment and flexibility.

You can't just go straight in and go, ‘I'm going to do a mile on a bike’, you wouldn't be able to do it, you'd cause yourself an injury, but go on the bike, say, ten minutes for the first time, then when you go the next time, go like a couple of more minutes. (Prostate cancer patient)

If they don't enjoy it, they won't carry on. (Breast cancer patient)

if, that you do back a [brand name] technology sort of based solution because then … you can put in those key dates and then it would reflect and sort of almost say ‘oh, you've had your treatment, it's time to rest now’ but offer perhaps like meditation or mindfulness at that point. So you're still doing something as part of your daily habit and then after the weeks or how long days of needing to, then it starts to reintroduce any exercise element … perhaps in a slightly different or more tailored to what we're going through at that time. (Charity representative)

Participants felt there could be improvements in both the patient's physical and mental well‐being from a social physical activity intervention but that patient engagement with a social intervention would vary according to personal preferences.

### 3.6 The All Together Active intervention

**FIGURE 1:**
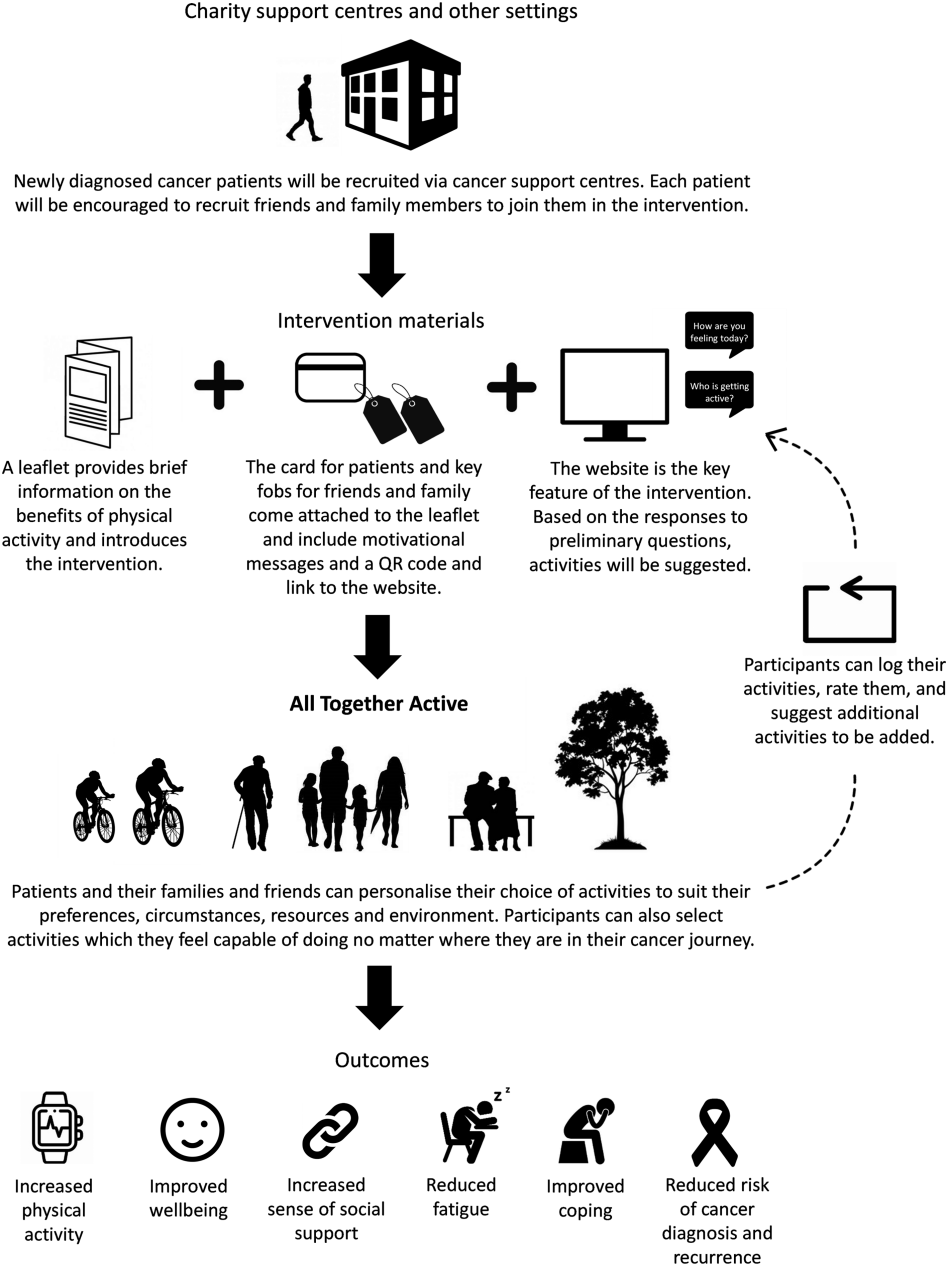
The All Together Active intervention pathway

Following the qualitative data collection and analysis, we discussed the research findings with the IDG and collectively developed the concept of All Together Active (Figure 1). The intervention consists of three components: an information leaflet, a ‘cue card’ with snap off key fobs and a website. Mock‐ups of all three resources have been produced, but the operational website is pending.

The leaflet provides information on the benefits of being active, addresses concerns that people may have about safety and includes a QR code for the website as well as the web address. The cue card includes short motivational messages for reinforcement, and the snap off key fobs, also including the QR code, are for family and friends to show their support for the patient through their engagement in All Together Active.

The website is the central feature of the intervention. A series of short opening questions about how the patient is feeling will be used to identify a selection of physical activities that might be appropriate for them and their family and friends. These range from low‐intensity and lifestyle activities through to moderate and vigorous physical activity options, which can all incorporate a social component. Patients will have the option to rate the appropriateness of any activities that they undertake for their particular stage of the cancer journey, as well as adding their own suggestions for enjoyable activities to the activity bank. The intervention will thus provide a community‐input forum around which the appropriateness of activities can be discussed by users. There will also be an option to sort and display activities according to rating. The website will have a forum for participants to offer support to others beyond their family and friend unit, as well as links and signposting to other community‐based initiatives that support people affected by cancer, and particularly those that support them to be physically active.

The intervention resources were co‐developed with the IDG to ensure they used appropriate language to encourage physical activity but at the same time not suggest blame or engender guilt in less active participants. The IDG selected activities to be included in the initial activity bank, graded by intensity, and therefore designed to be flexible and adaptable to people's stage of treatment and recovery, as well as their physical and mental health. We envisage the intervention being publicised online as well as at venues visited by those with cancer and their family and friends so that it is accessible to all, when it is the ‘right time’ for them.

## 4 DISCUSSION

Previous research has established that a cancer diagnosis negatively impacts the person with cancer and their family (Humpel et al., 2007; Kent et al., 2016) but can present a teachable moment for behaviour change (Frazelle & Friend, 2016; McBride et al., 2017; Radecki Breitkopf et al., 2014). It therefore represents an important opportunity for both secondary and primary cancer prevention through initiation of lifestyle interventions targeted at the person with cancer and their family and friends. All Together Active builds upon distinct foundational literatures in cancer, physical activity and family support and relationships (Gao et al., 2020). We believe our study is the first to explore how a social intervention could be formulated to optimise opportunities for physical activity behaviour change, based on the experiences of people recently diagnosed with breast or prostate cancer, their family and friends, as well as healthcare professionals and stakeholders from third sector physical activity and cancer organisations. In particular, we believe our underutilised approach in forming a collaborative IDG and in engaging with key stakeholders in the design of the intervention maximises the utility of the intervention but also helps in ensuring the support of community and clinical partners that play crucial roles in promoting available resources. Furthermore, we believe this approach provides a useful model for the collaborative development of other public health interventions for cancer survivors and their families and for individuals and families affected by other clinical conditions.

Recent cancer research has not only identified physical activity programmes and opportunities as an unmet need for people living with cancer (Mead et al., 2020) but has also found current online information regarding physical activity for cancer survivors to be lacking in specificity and safe suggestions for both engaging in physical activity and avoiding sedentary behaviour (Evans et al., 2021; Goodwin et al., 2020; Wong et al., 2018).

In our interviews, several key factors emerged that are likely to be vital considerations for effective interventions. These included: the timing of the intervention, which may be the point of diagnosis for some but post‐treatment for others; the intervention format and the need to tailor to different target groups including different levels of digital literacy; the framing of the message to avoid blame and guilt and emphasise positive benefits; the need for varied activities to encompass the very ill as well as the very fit; and the importance of flexibility to recognise that participants may be on different stages of their cancer journey. Many of our findings are consistent with what has been reported in other recent research into the facilitators and barriers to physical activity among cancer patients (Clifford et al., 2018).

The intervention developed from this research, All Together Active, is a flexible programme that addresses these considerations. It is designed to be accessed at any point along the cancer journey and the activities are varied, flexible and progressive. Importantly, the development of the intervention was informed by cancer patients and their family and friends. We therefore believe it is appropriate for these population groups and the language used is accessible and acceptable.

When developing All Together Active, it was envisaged that people would first find out about the intervention via cancer support centres or other physical venues. This aspect would likely be broadened in the context of COVID‐19. People living with cancer are at high risk of contracting infectious diseases and are being encouraged to stay at home shielding. However, the unintended consequences include negative impacts on physical and mental health and lack of access to in‐person community and clinical resources (Nekhlyudov et al., 2020). As such, there is greater reliance on online information and resources. Indeed, we believe the format of All Together Active is particularly well suited to the current public health context and social distancing regulations (American Psychological Association, 2020; Nekhlyudov et al., 2020; Sepúlveda‐Loyola et al., 2020). Signposting to All Together Active from cancer support websites or from government or other websites with information on staying active will broaden the reach of the intervention and provide a flexible and robust means to achieve uptake.

This paper illustrates the use and benefit of participatory methods, based on a co‐development approach, to design a public health intervention for cancer patients and their family and friends. Such approaches have the potential to bring substantial benefit as they allow a wide variety of stakeholders to provide input into the intervention design and therefore help to ensure that interventions are both deliverable and consummate of the requirements of those who will take part. The use of co‐development to ensure intervention fit not only maximises the chance of subsequent effectiveness but is also likely to widen participation and thus help reduce inequalities. A systematic review and meta‐analysis showed how community engagement is pivotal to addressing inequalities by enhancing buy‐in from the intervention's target populations (O'Mara‐Eves et al., 2013). Key to successful engagement is undoubtedly the involvement of appropriate groups of stakeholders, particularly given the evidence that the synergies that can develop from groupings of shared understanding can bring considerable learnings (Ong & Uddin, 2020). The tailoring that can arise from this form of engagement may also be particularly critical in condition‐specific interventions such as All Together Active where research teams involved in intervention design may have little experience of living with cancer themselves, thus limiting the quality of knowledge among those developing the intervention in the absence of participatory approaches (Foster et al., 2018). It was our stakeholders who ensured that the intervention is flexible enough in its content and delivery to reflect the considerable diversity of individuals' experiences of cancer. As illustrated by quotes above, they specified the need for a wide range of recommended activities of all intensities, which could be undertaken alone or collectively, and which reflect motivations from social activities to simply feel better, to exercise challenges that provide a focus for physical training. In doing so, they have ensured that the intervention can benefit those at different stages of their cancer journey and also allows for temporal variations in how well people feel and their capacity and motivation to be active during treatment or recovery. Stakeholders also highlighted the need for multiple delivery mediums including hard copy resources and an online platform to accommodate a diverse range of people who may vary in their preferences for accessing the intervention. Had we not engaged stakeholders with lived experience of cancer, these features would likely not have been considered, resulting in an intervention which may have excluded many and possibly only benefited a minority.

This study has several limitations. We only included patients diagnosed with breast or prostate cancer. We selected these specific types of cancer as they are the most common among females and males respectively and also cancer sites with some of the strongest evidence in terms of the health effects of physical activity. We recognise that the findings may not be generalisable to all cancer types. In addition, some participants felt unable to join the IDG, and as such, this group was formed of individuals who, when invited, were well enough to contribute, and perhaps more likely to consider being physically active. For some of these individuals, the data are based on retrospective accounts of experiences. While the sample size of 37 was large, the number of representatives from each stakeholder group was relatively small. However, we are confident in the breadth of input received, including the lived experience of different stages of the cancer journey. We also continued to interview people until we felt we had reached data saturation. Finally, the study was conducted in the context of the UK healthcare system and social structures. It is not known the degree to which the results and the intervention are generalisable to other contexts.

Nevertheless, we believe the study has several notable strengths including engagement with a wide range of stakeholders, the adoption of a rigorous methodology in order to identify key themes from discussions and the use of a strong interdisciplinary team with clinical and non‐clinical members bringing expertise in cancer management and treatment, intervention development and design, physical activity, the psychology of behaviour change and qualitative research methods. In addition, the use of a co‐design methodology meant that the intervention is more likely to be adopted by the target populations than more top‐down approaches.

Once the full website is developed, the next stage will be to undertake a feasibility study, followed by a definitive trial of the All Together Active intervention. This will provide insight into appropriate channels for advertisement and recruitment, the reach of the intervention, engagement in the online platform, cost‐effectiveness and the impact of the intervention on physical activity and other outcomes among cancer patients and those that support them.

## 5 CONCLUSION

All Together Active is a social intervention to support people recently affected by a cancer diagnosis to be physically active together. It was developed using a co‐design approach, which highlighted key learning with broad relevance to the development of public health interventions. Our findings suggest that interventions need to be flexible in terms of timing and delivery and offer a wide range of physical activity options. Avoidance of guilt and blame is a key consideration, especially if patients were not previously physically active. All Together Active is designed to support cancer patients and their family and friends to be active throughout treatment and beyond, benefitting their physical and mental health.

## CONFLICT OF INTEREST

All authors declare that they have no competing interests.

## AUTHOR CONTRIBUTIONS

All authors contributed to the study conception and design. SG collected the data. KP, SG and AC undertook the data analysis. KM led the preparation of the draft manuscript. All authors contributed to revising the manuscript and approved the final version.

## ETHICS STATEMENT

The project received ethical approval from the University of Surrey Research Ethics Committee (UEC 2019 109FHMS).

## Data Availability

Data available on request due to privacy/ethical restrictions.
